# Podoplanin-positive extracellular vesicles in ovarian cancer: linking thrombosis, platelet crosstalk, and cancer stemness – a narrative review

**DOI:** 10.1097/MS9.0000000000005293

**Published:** 2026-07-15

**Authors:** Shreya Singh Beniwal, Rovena Tali, Samid Soeb Munshi, Yash Satish Caroicar, Zainab, Dina Mohamed, Aala Alhamsa, Yujin Jeong, Prashasti Dahiya, Rafael Everton Assunção Ribeiro da Costa, Chimuka Mwaanga, Aarushi Mishra

**Affiliations:** a Lady Hardinge Medical College, New Delhi, India; b University of Medicine, Tirana, Albania; c B.J. Medical College, Ahmedabad, Gujarat, India; d Goa Medical Collegeand Hospital, Goa University, Goa, India; e Dr. VRK Women’s Medical College, Moinabad, Hyderabad, India; f University of Medical Sciences and Technology, Kigali, Rwanda; g Faculty of Medicine, University of Khartoum, Khartoum, Sudan; h Icahn School of Medicine at Mount Sinai/Elmhurst Hospital Center, Elmhurst, NY, USA; i ESIC Medical College and Hospital, Faridabad, India; j Universidade Estadual de Campinas (UNICAMP), Campus Cidade Universitária Zeferino Vaz, Campinas, Brazil; k Texila American University Zambia, Lusaka, Zambia; l Lvivs’kyj nacionaľ’nyj medychnyj universytet imeni Danyla Halyc’koho, Ukraine

**Keywords:** cancer stemness, extracellular vesicles, ovarian cancer, podoplanin, thrombosis

## Abstract

Ovarian cancer remains a leading cause of gynecologic cancer death worldwide, largely due to late diagnosis, frequent recurrence, and metastatic tendencies. Thrombosis is a common and life-threatening complication in these patients, contributing to poor prognosis and therapy resistance. Emerging evidence highlights a mechanistic link between tumor-derived extracellular vesicles and thrombotic events – particularly podoplanin-positive small extracellular vesicles (PDPN⁺ sEVs). Secreted by PDPN-expressing ovarian tumor cells, these vesicles act as biologically active messengers that circulate systemically. A central mechanism involves the binding of PDPN⁺ sEVs to C-type lectin-like receptor 2 (CLEC-2) on platelets, inducing platelet activation, aggregation, and the release of pro-inflammatory mediators. This interaction creates a hypercoagulable and pro-inflammatory microenvironment. Beyond coagulation, PDPN⁺ sEVs promote cancer aggressiveness by enhancing cancer stem cell plasticity, driving epithelial-to-mesenchymal transition, and facilitating immune evasion – hallmarks of metastasis and chemoresistance. This dual activity establishes a thromboinflammatory tumor niche that accelerates disease progression while undermining treatment efficacy. The ability of PDPN⁺ sEVs to circulate in ascitic fluid and peripheral blood positions them as promising candidates for liquid biopsy–based diagnostics. Furthermore, targeting the PDPN–CLEC‑2 axis or disrupting sEV biogenesis offers a novel therapeutic strategy to curb both thrombosis and metastatic spread. In conclusion, PDPN⁺ sEVs represent a critical molecular link between coagulation and cancer progression, offering valuable diagnostic, prognostic, and therapeutic potential for improving outcomes in ovarian cancer.

## Introduction

According to the World Health Organization’s (WHO) 2019 estimates, among causes of death before the age of 70 years, cancer ranks first or second in 112 out of 183 countries and third or fourth in another 23^[^1^]^. Ovarian carcinoma is the deadliest type of gynecologic cancer, responsible for more than 180 000 deaths globally each year^[^2^]^. It is the eighth most common cancer affecting women globally, accounting for about 3.7% of all cancer cases and 4.7% of cancer-related deaths in 2020^[^3^]^. The overall 5-year survival rate for patients with advanced epithelial ovarian cancer (EOC) is below 25%, primarily because the disease is often diagnosed at a late stage, with approximately 70% of cases identified at stages III or IV^[^4,5^]^. Outcomes for patients with advanced OC have shown little improvement in recent decades^[^6^]^. Recurrence occurs in nearly 25% of early-stage OC cases and in over 80% of those with stage III or IV. The initial recurrence is typically classified as platinum-sensitive or platinum-resistant, based on a 6-month platinum-free interval, which reflects the tumor’s underlying biological behavior and clinical response^[^7^]^.

Several factors contribute to the poor prognosis of OC, such as paraneoplastic thrombosis. Around one-third of women newly diagnosed with OC present with elevated platelet counts exceeding 450 000/µL^[^8^]^. In addition to clinical observations, experimental studies indicate that platelets actively contribute to cancer progression through various mechanisms, such as shielding cancer cells from immune detection, facilitating their arrest within the microvasculature, and promoting angiogenesis^[^9^]^.

Among the molecular interactions involved, the binding of tumor-expressed podoplanin (PDPN) to platelet C-type lectin-like receptor 2 (CLEC-2) has emerged as a key focus of investigation. CLEC-2, encoded by the CLEC1B gene on chromosome 12, is a member of the Dectin-1 cluster within the family of C-type lectin-like immune receptors. It functions as a type II transmembrane protein featuring a C-terminal extracellular domain that notably lacks the conserved amino acid residues typically required for carbohydrate binding, distinguishing it structurally from classical carbohydrate-recognizing lectins^[^10^]^. PDPN is a transmembrane glycoprotein that is overexpressed in various human malignancies, particularly in tumors originating from stratified squamous epithelium (SCCs). Its presence in cancer cells is associated with enhanced migratory capacity and increased invasive behavior^[^11^]^. The interaction between PDPN and CLEC-2 occurs specifically at the PLAG3 region within the heavily O-glycosylated extracellular domain (ectodomain) of PDPN. CLEC-2, found on platelet cells, binds to this PLAG3 domain of PDPN, and this binding activates platelet aggregation^[^12^]^. In experimental models, blocking the PDPN–CLEC-2 interaction using the monoclonal antibody NZ-1 significantly reduced platelet aggregation and suppressed lung metastasis, highlighting the interaction’s pathophysiological importance and therapeutic potential in cancer metastasis^[^13^]^. Although the role of thrombocytosis in OC progression is not yet fully understood, previous experimental studies have associated it with response to chemotherapy. These studies suggest that combining taxane-based treatments with platelet-depleting antibodies can significantly reduce tumor burden^[^14^]^.

Another critical factor contributing to OC recurrence is intratumor heterogeneity and cancer stem cells (CSCs). OC is commonly categorized into five major subtypes according to their distinctive histopathological features: (1) high-grade serous carcinoma (HGSC); (2) low-grade serous carcinoma (LGSC); (3) mucinous carcinoma (MC); (4) endometrioid carcinoma (EC); and (5) clear cell carcinoma (CCC)^[^15^]^. The primary clinical implication of ovarian tumor heterogeneity is that molecularly targeted therapies may demonstrate efficacy at certain tumor sites but fail at others, thereby limiting their overall therapeutic impact. This variability in response likely contributes to the suboptimal outcomes observed with targeted treatments in EOC. Consequently, assessing the degree of tumor heterogeneity may be as critical as staging in the pre-treatment evaluation of EOC patients^[^16^]^. CSCs are genetically altered cells whose frequency varies by tumor type – they can be either scarce or relatively widespread. They contribute to tumor heterogeneity by maintaining their capacity for self-renewal and their ability to differentiate into multiple cell lineages^[^17^]^. Previous studies have demonstrated that OC stem cells play a pivotal role in the formation of multicellular spheroids during the transcoelomic peritoneal dissemination of OC^[^5^]^.

Several markers used to identify normal stem cells, such as CD133, also help isolate CSCs across various tumors. While CD133’s exact function is unclear, it marks both hematopoietic and endothelial progenitor cells, which may contribute to tumor vasculature. Aldehyde dehydrogenase enzymes are linked to chemotherapy resistance via drug metabolism and oxidative stress regulation. Small tumor-initiating stem cells found in ovarian tissue show distinct gene expression, including high levels of SOX17, FOXQ1, and MUC16/CA125. These cells may originate from dormant embryonic-like stem cells that, under certain conditions, transform into active CSCs driving tumor growth^[^18–20^]^.

HIGHLIGHTS

- Podoplanin-positive extracellular vesicles (PDPN⁺ sEVs) link ovarian cancer progression to thrombosis and immune modulation.
- PDPN⁺ sEVs interact with CLEC-2 on platelets, driving platelet activation, aggregation, and thromboinflammation.
- These vesicles enhance cancer stemness, EMT, and immune evasion, promoting metastasis and therapy resistance.
- Circulating PDPN⁺ sEVs in ascitic fluid and blood show strong potential as liquid biopsy biomarkers.
- Targeting the PDPN–CLEC-2 signaling axis offers a promising dual strategy to reduce both thrombosis and tumor aggressiveness.

**Figure 1.**
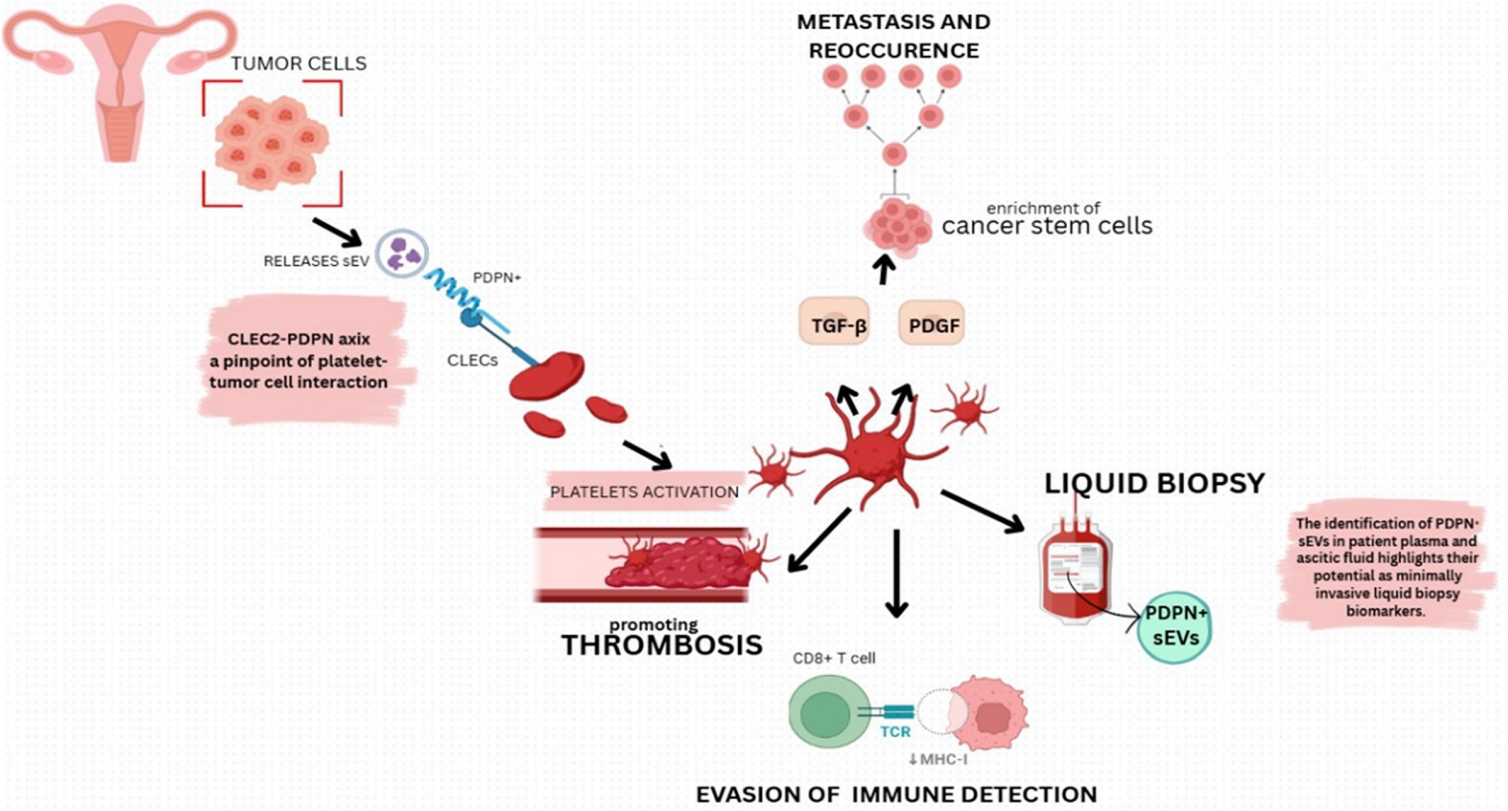
Podoplanin-positive small extracellular vesicles (PDPN⁺ sEVs) as mediators of thromboinflammation and cancer stemness in ovarian cancer. Tumor-derived PDPN⁺ sEVs bind to CLEC-2 receptors on platelets, triggering platelet activation, aggregation, and the release of pro-inflammatory and pro-stemness mediators such as TGF-β. This signaling cascade promotes a hypercoagulable microenvironment, epithelial–mesenchymal transition (EMT), immune evasion, and the enrichment of cancer stem cell traits, facilitating metastatic progression^[^5,18–20^]^.

Another emerging factor contributing to OC progression and recurrence is the role of extracellular vesicles (EVs). EVs are small, spherical, membrane-bound particles released into the extracellular space, capable of transporting biologically active molecules to both nearby and distant cells^[^21^]^. Their significance lies in their ability to transfer functional information that can influence the behavior of recipient cells in OC^[^22^]^. EVs play a key role in mediating communication between cancer cells and their surrounding environment, regulating survival and signaling pathways. This interaction contributes to the development of resistance to various cancer therapies^[^23^]^. EVs carry a diverse cargo of biological molecules, and growing evidence suggests they play a crucial role in drug resistance. For instance, cisplatin-resistant OC cells release EVs at a rate 2.6 times higher than that of drug-sensitive cells^[^24^]^. Lastly, the interplay between PDPN⁺ small EVs (sEVs), platelet activation, and cancer progression is schematically illustrated in Figure 1^[^5,18–20^]^.

This narrative review aims to elucidate the mechanistic role of PDPN-positive EVs (PDPN⁺ sEVs) in linking thrombosis, platelet activation, and CSC plasticity in OC. Furthermore, it discusses the translational relevance of the PDPN–CLEC-2 axis and EV-associated non-coding RNAs as emerging biomarkers and therapeutic targets for improving risk stratification, treatment response, and clinical outcomes in OC^[^5,9,12,25^]^.

As a narrative review, this manuscript is based on a comprehensive, non-systematic appraisal of the literature. Articles were identified through searches of PubMed, Scopus, and Web of Science using combinations of the terms “ovarian cancer,” “podoplanin,” “CLEC-2,” “extracellular vesicles,” “platelets,” “thrombosis,” and “cancer stem cells.” Priority was given to original experimental studies, translational research, and clinically relevant reviews published predominantly between 2010 and 2025. Reference lists of key articles were also screened to ensure coverage of seminal and mechanistically relevant studies^[^1–3,9,12^]^.

## Podoplanin and CLEC-2 axis: cancer-platelet crosstalk

### Structure, expression, and regulation of PDPN in tumors

PDPN is a 43-kDa mucin-type transmembrane glycoprotein that comprises an extracellular domain rich in serine/threonine, which serves as potential O-glycosylation sites and facilitates interactions with platelet receptors, a singular transmembrane segment, and a short cytoplasmic tail. It is present on kidney podocytes, where it is postulated to be involved in modulating the morphology of podocytes and the functionality of the glomerular filtration barrier^[^25,26^]^. Importantly, PDPN functions as an endogenous ligand for the platelet receptor CLEC-2^[^26^]^.

Physiologically, PDPN is expressed in type 1 alveolar cells in the lungs, fibroblastic reticular cells in the lymph nodes, synovial cells in the joints, and lymphatic endothelial cells, but not in vascular endothelial cells^[^27^]^. Under malignant transformation, PDPN is aberrantly upregulated in tumor cells – including squamous cell cancer, seminomas, osteosarcoma, glioblastoma, and ovarian germ cell tumors^[^28,29^]^ – as well as in the tumor stroma, notably in cancer-associated fibroblasts (CAFs)^[^30^]^. In epithelial ovarian carcinomas, PDPN expression is correlated with clear cell histology and has been associated with advanced tumor stage and aggressiveness^[^25^]^. PDPN’s dual localization in both tumor cells and CAFs underscores its multifaceted role in the TME. In tumor cells, PDPN contributes to motility and invasion; whereas, in CAFs, PDPN⁺ subsets may participate in extracellular matrix remodeling, immune suppression, and metastatic niche preparation^[^30^]^.

### CLEC-2 receptor biology and platelet activation

CLEC-2 is a 32–40 kDa type II membrane protein containing one C-type lectin-like domain^[^31^]^. First recognized in 2006 as a receptor for the snake venom toxin rhodocytin, CLEC-2 has now been characterized as a central mediator of platelet activation via interaction with its endogenous ligand, PDPN^[^32^]^. CLEC-2 is almost exclusively expressed in platelets and megakaryocytes, with minimal expression in other cell types, such as Kupffer cells^[^27^]^, making it an attractive target for anti-metastatic therapy with limited off-target effects.

**Figure 2.**
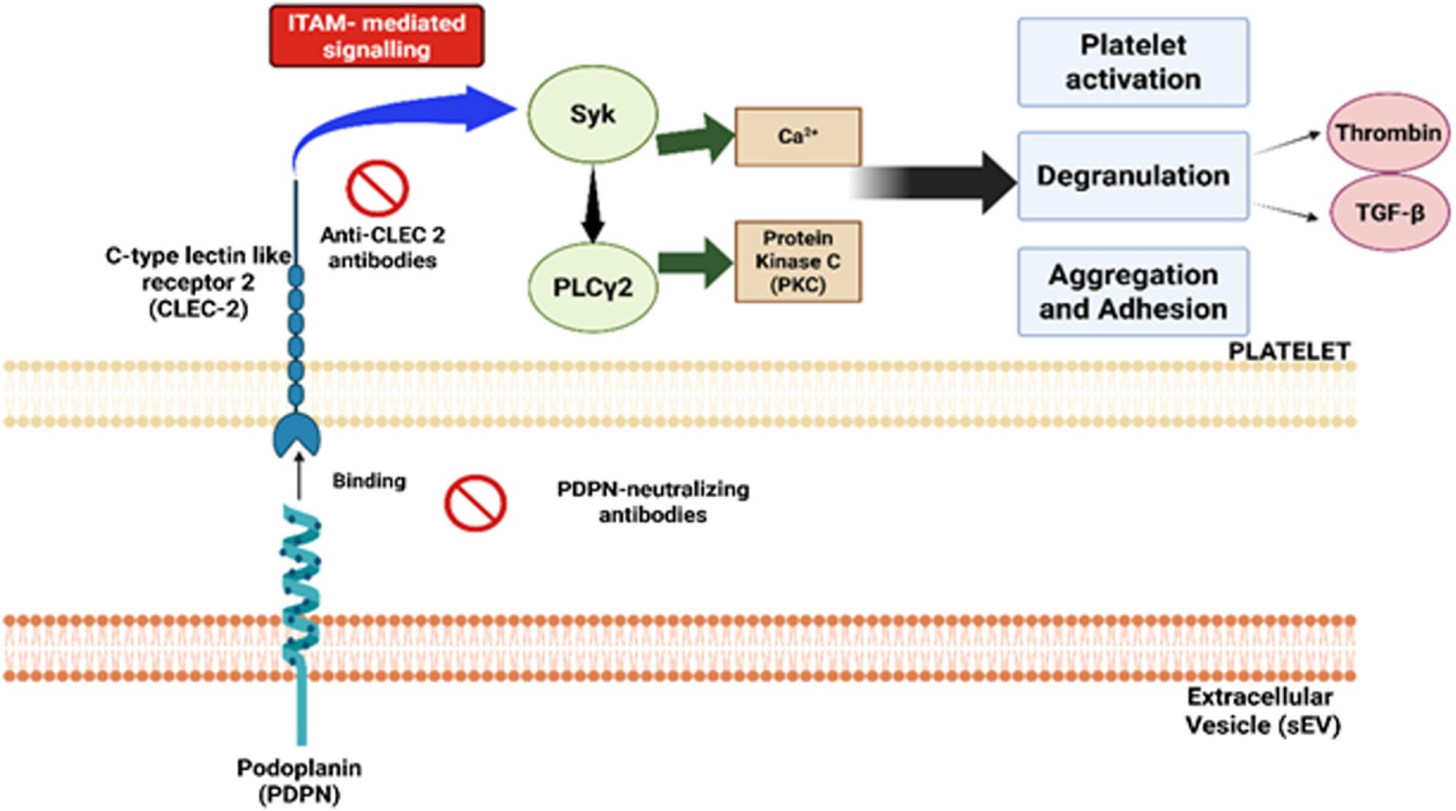
Molecular mechanism of PDPN–CLEC-2–mediated platelet activation. PDPN expressed on tumor-derived sEVs engages CLEC-2 on platelets, activating hemITAM-dependent signaling via Src, Syk, and PLCγ2. This leads to calcium mobilization, PKC activation, and platelet degranulation with the release of thrombin and TGF-β, reinforcing thrombosis, immune evasion, and metastatic competence^[^12,25,35,37^]^.

Upon PDPN or rhodocytin binding, CLEC-2 undergoes tyrosine phosphorylation at its hemITAM (YXXL) motif by Src family kinases. This recruits Syk tyrosine kinase via SH2 domains, triggering a downstream signaling cascade involving the adaptor proteins LAT and SLP-76, and the effector enzyme phospholipase Cγ2 (PLCγ2)^[^12^]^. This axis facilitates intracellular calcium mobilization and protein kinase C activation, culminating in platelet activation and aggregation^[^33^]^. Furthermore, the molecular mechanism by which PDPN⁺ sEVs activate CLEC-2 signaling and promote platelet aggregation is illustrated in Figure 2.

This activation cascade promotes the secretion of granules containing pro-tumorigenic factors, such as thrombin and TGF-β, contributing to a tumor-supportive microenvironment that facilitates immune evasion, angiogenesis, and metastasis^[^34^]^.

### Functional role of PDPN-CLEC-2 in thrombosis and tumor metastasis

Platelet-tumor interactions are frequently utilized by tumor cells to aid in their survival and spread. Circulating tumor cells are protected from shear stress and immune surveillance, especially NK cell-mediated cytotoxicity, by platelet aggregates that envelop them. Models of melanoma and OC have demonstrated this phenomenon^[^25,35^]^.

In preclinical murine models of ovarian and melanoma cancer, PDPN–CLEC-2–mediated platelet activation enhances metastatic dissemination, whereas genetic deletion or antibody-mediated blockade of CLEC-2 significantly reduces thrombus formation and metastatic burden^[^25,33,35,36^]^. The clinical significance of this signaling axis is highlighted by the fact that, *in vivo*, CLEC-2 knockout or platelet-specific deletion of CLEC-2 leads to decreased thrombus formation, decreased metastatic burden, and altered immune cell infiltration in tumor-bearing models^[^35,36^]^.

## PDPN⁺ extracellular vesicles in ovarian cancer

The sEVs, including exosomes and microvesicles, are lipid bilayer-bound particles released by cells that mediate intercellular communication and are abundant in the TME of OC^[^38^]^. Tumor-derived sEVs promote cancer progression by transferring oncogenic proteins, nucleic acids, and lipids to recipient cells^[^39^]^. While the biological roles of exosomes and microvesicles have been widely investigated, the functions of larger atypical EVs–namely, large oncosomes (LOs)–are still emerging. LOs, ranging from 1 to 10 µm in size, are released by highly migratory, amoeboid tumor cells and may serve as vehicles for active biomolecules, including PDPN^[^39,40^]^.

PDPN is a type I transmembrane glycoprotein implicated in cell migration, platelet aggregation, and lymphangiogenesis^[^41,42^]^. In OC, PDPN expression is upregulated and has been linked to peritoneal dissemination and metastasis^[^41–43^]^. PDPN⁺ EVs–particularly those derived from highly invasive OC cells–have been identified as mediators of tumor progression by promoting a pro-thrombotic state and modulating immune responses^[^43,44^]^. PDPN binds to CLEC-2 on platelets, triggering their aggregation and facilitating the hematogenous spread of tumor cells^[^45^]^. This interaction has also been shown to enhance platelet-mediated immune evasion and metastasis in murine models^[^45–47^]^.

LOs enriched in PDPN further contribute to these effects by serving as decoys for CLEC-2, thereby promoting widespread platelet activation in circulation^[^48^]^. PDPN⁺ EVs can be captured by CD169⁺ macrophages in lymph nodes, influencing antigen presentation and shaping immune tolerance^[^49^]^. In OC patients, exosome-mediated thrombin generation has been correlated with disease progression and poor prognosis^[^50^]^. Targeting the PDPN-CLEC-2 axis has shown promise in reducing thrombosis and metastatic burden in preclinical models, suggesting its therapeutic relevance^[^51^]^.

Beyond thrombosis, PDPN⁺ sEVs are involved in remodeling the TME. PDPN expression modulates EMT and interacts with CD44 to promote directional cell migration^[^52,53^]^. The redistribution of PDPN in migrating cells and its enrichment in EVs highlight its potential role in dynamic TME interactions^[^54,55^]^. Moreover, PDPN signaling in tumor-derived EVs can activate stromal fibroblasts and endothelial cells, further fostering angiogenesis and matrix remodeling^[^56^]^.

Therapeutic strategies aiming to neutralize PDPN⁺ EVs – using monoclonal antibodies or CLEC-2 mimetics – are under development to reduce tumor-associated thrombo-inflammation and metastasis^[^57^]^. Additionally, PDPN⁺ EVs represent promising diagnostic biomarkers due to their stability in circulation and specific association with aggressive OC phenotypes^[^58^]^. Despite growing interest, the full spectrum of PDPN⁺ EV functions–especially large oncosome-mediated interactions with the TME–remains underexplored^[^39,59^]^.

## Platelets, sEVs, and cancer stem cell plasticity

### Platelet-derived factors that promote EMT and stemness

Platelets have long been known to play hemostatic and thrombo-inflammatory roles, but they also play an active role in orchestrating tumor cell plasticity and metastasis by releasing important growth factors and vesicular cargo upon activation by tumor-derived stimuli^[^60,61^]^. Ovarian and other solid tumors receive both platelet and tumor cell contributions of increased levels of TGF-β and platelet-derived growth factor (PDGF), but the platelet α-granules are a highly concentrated source of these drivers of EMT^[^62^]^. Research has linked the production of CSCs to EMT activation^[^63^]^. The loss of epithelial markers and the acquisition of mesenchymal markers characteristic of “cancer stem-like cells,” which lead to increased cell invasion and metastasis *in vivo*, are characteristics of EMT^[^60,64^]^. Numerous studies have documented the acquisition of stemness in a variety of cancer types, including ovarian, colorectal, prostate, and pancreatic cancer, after an EMT program is activated^[^65–68^]^.

TGF-β activates SMAD-dependent signaling to suppress E-cadherin and induce vimentin, promoting a mesenchymal phenotype, whereas PDGF synergizes through MAPK and PI3K/AKT activation to promote proliferation and invasive potential^[^64^]^. Although initial EMT induction by the axis is transient, prolonged TGF-β/PDGF exposure – frequent in reactive thrombocytosis – tends to fix stable stem-like characteristics, underpinning long-term CSC plasticity^[^69^]^. Notably, PDGF receptor A (PDGFR) inhibitors ripretinib, avapritinib, and crenolanib are being explored in clinical trials to break this EMT–stemness loop and sensitize tumors^[^70^]^.

Reactive thrombocytosis in OC is primarily tumor-secreted interleukin-6 (IL-6)–driven, increasing thrombopoietin (TPO) production and megakaryopoiesis, with a correlation to poor prognosis^[^9,71^]^. Activated platelets release platelet-derived microparticles (PMPs) – which account for up to 90% of the circulating micro particles (MPs) – rich in TGF-β, PDGF, miRNAs, and immunomodulatory surface proteins^[^72^]^. PMPs have been extensively characterized in OC models by nanoparticle tracking analysis and flow cytometry and have been demonstrated to induce EMT, chemoresistance, and CSC features *in vitro* and *in vivo*^[^73–76^]^. Due to their strong optical and magnetic responses and high biocompatibility, gold nanoparticles (Au NPs) have become a promising tool for the diagnosis and treatment of OC^[^77^]^.

Beyond their pro-thrombotic role, platelet-derived signals converge on pathways regulating tumor plasticity, establishing a direct mechanistic link between coagulation, epithelial–mesenchymal transition, and CSC maintenance^[^60–65^]^.

### PDPN⁺ EVs and platelet-mediated CSC reprogramming

**Figure 3.**
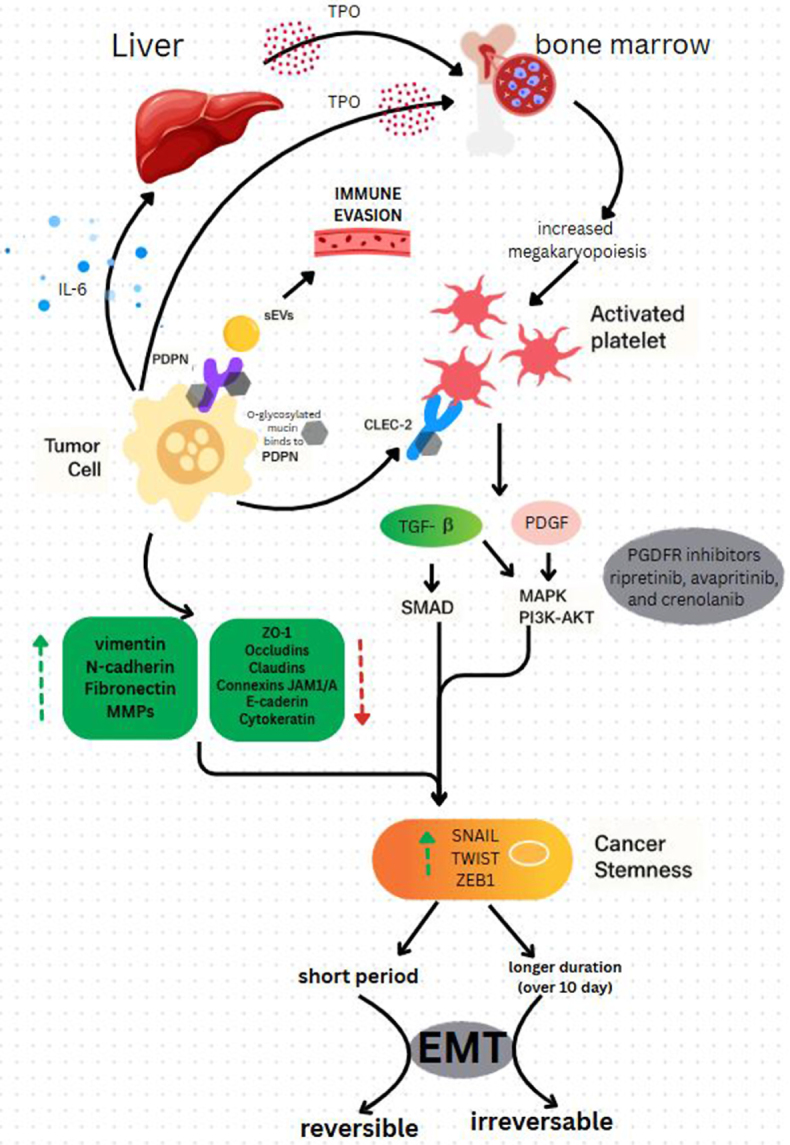
PDPN⁺ sEV–platelet crosstalk drives EMT and cancer stem cell plasticity. Tumor-derived IL-6 promotes thrombopoiesis, while PDPN–CLEC-2 interaction induces platelet activation and the release of sEVs enriched in TGF-β, PDGF, and non-coding RNAs. These factors activate SMAD and PI3K–AKT signaling pathways, upregulating EMT-associated transcription factors (SNAIL, TWIST, ZEB1) and stabilizing cancer stem-like phenotypes^[^9,30,52,64^]^.

PDPN, a highly O-glycosylated mucin expressed on tumor and stromal cells, binds to the CLEC-2 receptor on platelets to trigger a feed-forward activation loop (Figure 3)^[^30,52,78,79^]^. PDPN⁺ EVs produced by tumors activate platelet degranulation, which releases additional TGF-β- and PDGF-enriched vesicles that enhance CSC programming^[^28,52,64,80^]^. Although imperfect EMT triggered by this loop is reversible, continuous signaling tends to result in the fixed acquisition of tumor-initiating properties through upregulated SNAIL, TWIST, and ZEB1 transcription factors^[^81^]^. They explain that cells stimulated for longer periods (10+ days) may render EMT irreversible and become “locked” in a mesenchymal state, while cells stimulated for shorter periods (2 to 6 days) may return to an epithelial state after the signal or stimulus is removed^[^82^]^.

Platelet sEVs transfer non-coding RNAs – most analyses to date employ primary patient platelet preparations, although some *in vitro* models have been instructive – especially miR-223 and oncogenic lncRNAs that reorganize chromatin^[^83–86^]^. For instance, miR-223 and miR-21 suppress tumor suppressors (e.g., PTEN), whereas lncRNAs regulate epigenetic marks like H3K27me3 through EZH2 (a histone methyltransferase) and augment DNA damage responses via CHK1 (a checkpoint kinase)^[^87–89^]^. These preserved mechanisms, reported in breast and lung cancer models, highlight the pan-tumor significance of platelet-EV-induced CSC reprogramming^[^73,85,90^]^.

### Platelet sEVs preserving the CSC niche and immune evasion

In addition to reprogramming tumor cells, platelet-derived sEVs shape a permissive CSC niche. CSCs are protected from external threats like immune attacks or therapeutic agents by this niche, which is made up of a diverse range of cell types such as immune cells, stromal cells, endothelial cells, pericytes, and fibroblasts, as well as cytokines, growth factors, metabolites, and extracellular matrix (ECM) components^[^85^]^. They transfer integrins (such as αIIbβ3), tissue factor, and bioactive lipids to promote angiogenesis, ECM remodeling, and vascular permeability^[^85^]^. PMPs can extravasate through leaky tumor neovasculature due to increased endothelial permeability from dysfunctional junctions – a hallmark of solid tumors. This permits PMPs to infiltrate the perivascular or stromal space within the TME and directly associate with tumor cells^[^91^]^. Antithrombotic agents, such as low-dose aspirin, have been experimentally demonstrated to reduce platelet-tumor interactions. Additionally, they can counteract platelets’ inhibitory effect on T cell-mediated cytotoxicity *in vitro*^[^91,92^]^.

Platelet cloaking of circulating tumor cells (CTCs) protects them from lysis mediated by natural killer (NK) cells; it has been imaged in patient-derived CTC clusters using confocal microscopy^[^92–94^]^. Various platelet-released factors – TGF-β, VEGF, 5-HT, PF4, and PGE2 – can – inhibit dendritic cell (DC) maturation and functional antigen presentation, promoting immune evasion in tumor environments^[^92^]^. Platelet P-selectin initiates cross-presentation and DC differentiation in blood monocytes^[^92^]^. Cumulatively, all these actions solidify a microenvironment that both maintains CSCs and evades host immunity.

## Exosomal noncoding RNAs in ovarian cancer progression

### Exosomal miRNAs in chemoresistance and EMT

Cancer cells selectively package specific miRNAs into exosomes, which are then released to modulate and reshape the surrounding TME^[^95^]^. These miRNAs play key roles in controlling tumor development and progression. In OC, exosomal miRNAs are increasingly recognized for their potential as biomarkers for early detection and disease monitoring. It has also been postulated that the higher release of these exosomes correlates with their higher invasive potential^[^96^]^. MiRNA-21 is of particular importance here, since it plays a pivotal role in cancer initiation, progression, as well as chemoresistance. It is overexpressed in most cancer types, including EOC^[^96,97^]^.

In a study comparing the expression patterns of exosomal miRNAs, miRNAs such as miR-16, miR-21, miR-93, miR-100, miR-126, miR-200b, miR-223, and miR-320 also showed altered expression in patients with EOC compared to healthy individuals. Additionally, the levels of exosomal miR-23a and miR-92a were linked to patients with ovarian cystadenoma^[^96^]^.

In another study comparing the expression of microRNAs in serum-derived exosomes among three different patient groups with ovarian masses, the expression levels of miR-93, miR-145, and miR-200c were found to be significantly elevated in serum-derived exosomes from OC patients (including both HGSOC and non-HGSOC subtypes) compared to those from non-cancer cases, such as benign tumors and borderline ovarian tumors (BOT)^[^98^]^.

Mo, Yulan *et al* (2023) studied the role of tumor-derived exosomal miR-141 in OC metastasis and found that this exosomal miRNA is abundantly secreted by OC cells and plays a key role in transforming stromal fibroblasts into proinflammatory CAFs, thereby promoting metastatic colonization^[^99^]^.

Yoshimura, Akihiko *et al* established that serum levels of miR-99a-5p are markedly increased in OC patients. Exosomal miR-99a-5p, derived from EOC cells, enhances cell invasion by modulating human peritoneal mesothelial cells (HPMCs) through the upregulation of fibronectin and vitronectin, suggesting its potential as a therapeutic target to impede OC progression^[^100^]^.

**Figure 4.**
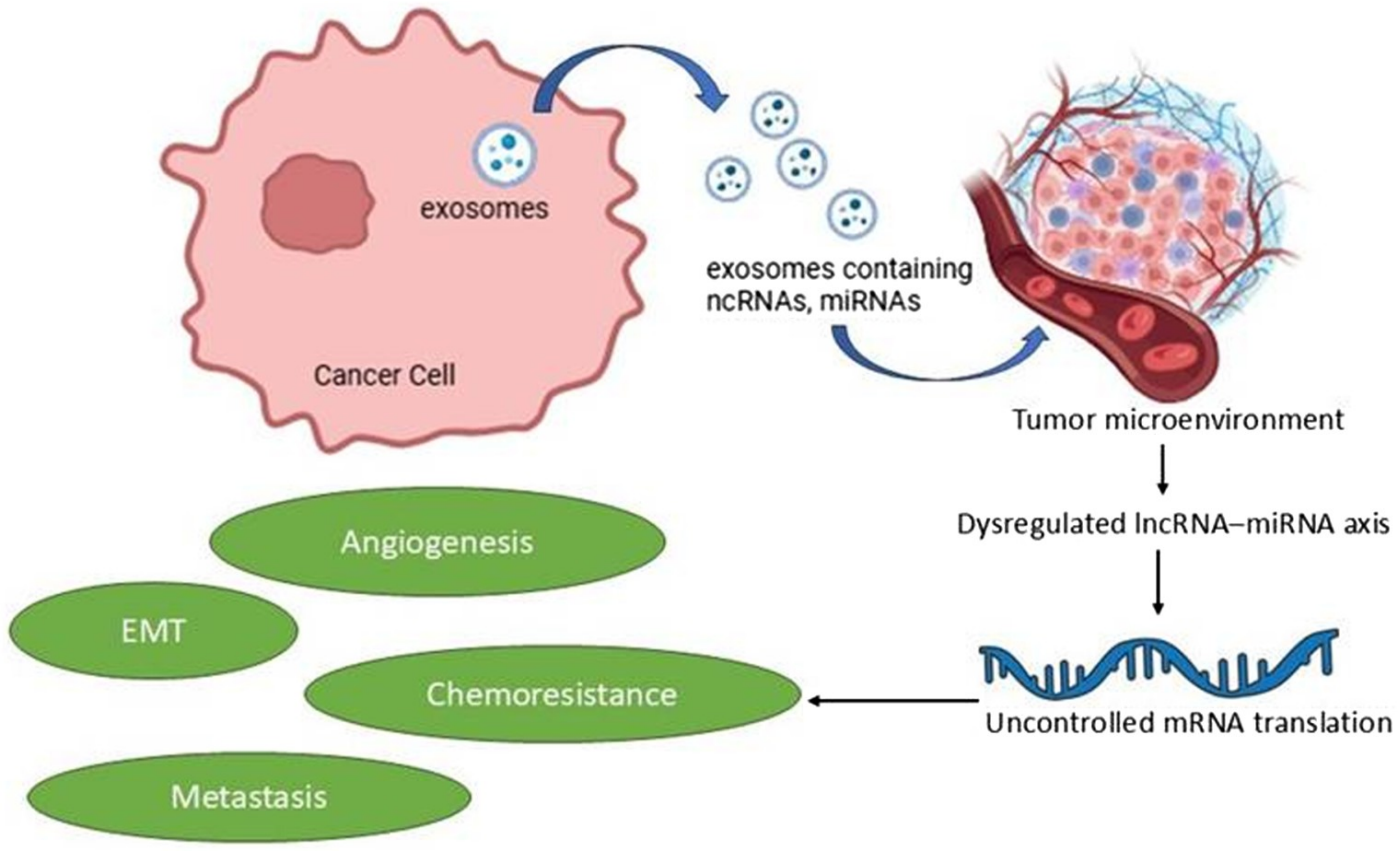
Exosomal ncRNAs promote cancer stemness and chemoresistance in ovarian cancer. The proposed mechanism by which ncRNAs influence ovarian cancer metastasis and chemoresistance involves the regulation of target genes and associated signaling pathways. Dysregulated RNA expression affects key processes such as epithelial-mesenchymal transition (EMT), cancer stem cell (CSC) maintenance, angiogenesis, apoptosis, cell cycle progression, and drug efflux, ultimately contributing to both the chemoresistance and metastatic potential of ovarian cancer cells. Created by authors based on data from^[^112,113^]^.

Some miRNAs, like miR-1246, have been linked to enhanced chemoresistance in OC. Targeting this pathway using miR-1246 inhibitors has been proposed as a promising therapeutic strategy to overcome drug resistance^[^95^]^. See Figure 4 for an overview of key exosomal miRNAs driving chemoresistance, EMT, and metastatic progression in OC.

### lncRNAs and circRNAs driving CSC traits and angiogenesis

LncRNAs, especially MALAT1, play significant roles in the onset as well as the progression of EOC. It contributes to the promotion of EMT by suppressing miR-22, a microRNA that plays a crucial role in preventing tumor malignancy through its inhibition of angiogenesis and cell cycle progression^[^101^]^. Other lncRNAs, such as lncRNA ASB16-AS1, SNHG20, AC005224.4, CACNA1G-AS1, PCAT6, PART1, etc., have been implicated in the development and progression of ovarian carcinoma^[^102–107^]^.

Recent studies have demonstrated that circular RNAs (circRNAs) play a role in the development and progression of OC. These act by functioning as a sponge for a tumor suppressor miRNA. One circRNA of importance is circ MYLK, which may contribute to the malignant progression of OC by modulating microRNA-652, and its expression has been significantly linked to advanced pathological stages and poor patient prognosis. Patients with elevated levels of circ MYLK expression tend to present with more advanced disease stages and reduced overall survival^[^108,109^]^. Other circRNAs, such as circRNA-UBAP2, circMUC16, circEPSTI1, etc., are also implicated in carcinogenesis^[^108^]^.

Besides these, CircCELSR1, circ_CELSR1, circATL2, circNRIP1, circTNPO3, and hsa_circ_0000714 are examples of circRNAs that play key roles in defining or influencing the chemoresistant characteristics of OC cells. Given that their expression levels impact how these cells respond to chemotherapy, they hold potential as biomarkers for tracking molecular treatment responses^[^108^]^.

### EV-packaged non-coding RNAs as stress-response regulators in hypoxia and therapy resistance

In response to hypoxic conditions within tumors, cancer cells can secrete soluble factors like VEGF and exosomes, which collectively drive abnormal blood vessel formation (pathological angiogenesis). Multiple studies have demonstrated that hypoxia influences the expression of various non-coding (nc) RNAs that are transported via exosomes. Recent research has also underscored the contribution of hypoxia-induced exosomes to both angiogenesis and tumor progression^[^110^]^. The ncRNAs are selectively packed into exosomes, and once transferred to recipient cells, these exosomal ncRNAs influence vital processes involved in tumor progression, including cancer initiation, metastasis, formation of new blood vessels, immune system modulation, and resistance to therapy. For instance, exosomes containing miR-1246 released by OC cells are taken up by other tumorigenic cells within the TME, thereby inducing resistance to paclitaxel chemotherapy through the suppression of CAV1^[^95,111^]^. Therefore, targeting exosomal ncRNAs holds potential for future clinical trials, either as standalone agents or in combination with existing treatments, to improve chemotherapeutic sensitivity in OC^[^111^]^.

## Clinical implications and therapeutic outlook

### Liquid biopsy: PDPN^+^ sEVs and ncRNA biomarkers for risk prediction

Liquid biopsy, harnessing PDPN-positive sEVs (PDPN^+^ sEVs) and non-coding RNAs (ncRNAs), transforms OC risk assessment by offering a non-invasive window into disease dynamics. PDPN^+^ sEVs, shed by OC cells into plasma, signal heightened risks of metastasis and thrombosis through their binding to CLEC-2 on platelets^[^9^]^. In high-grade serous OC, elevated PDPN^+^ sEV levels often herald poor prognosis, tied to their role in fueling cancer stemness and drug resistance^[^114^]^. ncRNAs, like miR-200b, carried within these vesicles, act as sensitive markers for tracking treatment response and forecasting progression-free survival^[^115^]^. By weaving PDPN^+^ sEV and ncRNA profiling into liquid biopsy frameworks, clinicians can sharpen early detection, risk stratification, and craft tailored treatments, especially for patients with advanced or recurrent OC, paving the way for more precise care. PDPN⁺ sEVs and ncRNA biomarkers are typically measured in plasma using ultra sensitive assays; elevated levels can indicate platinum resistance, prompting earlier therapeutic shifts.

### Targeting PDPN–CLEC-2 axis and sEV biogenesis

Disrupting the PDPN–CLEC-2 axis and sEV biogenesis offers an opportunity for tackling OC and its thrombotic complications. The PDPN–CLEC-2 interaction spurs tumor cell-induced platelet aggregation (TCIPA), driving metastasis and chemo resistance^[^9^]^. A novel 5-nitrobenzoate, 2CP, blocks this axis, curbing TCIPA and metastasis without raising bleeding risks, and boosts cisplatin’s punch in preclinical models^[^78^]^. Meanwhile, drugs like GW4869, which target neutral sphingomyelinase, dial down sEV biogenesis, slashing PDPN^+^ sEV release and weakening tumor-supportive pathways^[^116^]^. Pairing these agents with mainstay treatments like carboplatin could shift the tide against platinum-resistant OC by targeting CSCs and the pro-thrombotic complications. These approaches demand rigorous clinical trials to confirm their promise, fine-tune dosing, and ensure safety, potentially reshaping how we confront the aggressive biology of OC and its deadly complications. Targeting the PDPN–CLEC-2 axis and sEV biogenesis remains largely preclinical, with early trials emerging. Safety concerns include bleeding risks and off-target toxicities due to platelet interaction.

### ncRNA inhibitors and EV-based delivery systems

The ncRNA inhibitors targeting miRNAs like miR-21 in PDPN^+^ sEVs hold potential to reverse chemo resistance in OC^[^116^]^. By using antisense oligonucleotides or siRNAs, delivered through engineered EVs, these inhibitors can silence resistance-driving miRNAs, restoring sensitivity to drugs like paclitaxel^[^117^]^. Engineered EVs exploit sEVs' natural affinity for tumor cells, ensuring precise, biocompatible delivery^[^118^]^. Early studies show these inhibitors shrink tumor and curb metastasis, hinting at their value in combination therapies. This strategy could redefine OC treatment by tackling resistance head-on, but clinical studies are needed to perfect the delivery and confirm efficacy in patients. Delivery systems under investigation include lipid-based nanoparticles, engineered EVs modified with tumor-targeting ligands, and, to a lesser extent, viral vectors, with engineered EVs showing superior biocompatibility and tumor specificity^[^119^]^. In animal models, miR-21 inhibitors delivered via engineered EVs have demonstrated significant tumor reduction in OC xenografts, with reduced tumor volume and metastatic spread compared to controls^[^120^]^. These findings strengthen the case for advancing EV-based ncRNA therapies into clinical trials.

### Integration into precision oncology trials

Incorporating PDPN^+^ sEV and ncRNA profiling into precision oncology trials sharpens patient selection and treatment design. Liquid biopsy assays tracking PDPN^+^ sEVs and miR-200b can steer patients toward trials targeting the PDPN–CLEC-2 axis or sEV biogenesis^[^115^]^. Pairing these biomarkers with therapies like PARP inhibitors boosts outcomes in BRCA-mutated or homologous recombination-deficient OC^[^121^]^. This approach tailors treatments to individual profiles, enhancing trial efficiency and patient outcomes. By identifying those most likely to respond, it paves the way for personalized medicine, addressing critical gaps in OC care and accelerating targeted therapy development. For example, a phase II trial design could select patients with high PDPN^+^ sEV levels for a combination therapy of 2CP and carboplatin, aiming to disrupt TCIPA and enhance chemotherapy efficacy^[^122^]^. This approach complements BRCA or HRD profiling by identifying actionable targets in BRCA-wildtype patients, where PDPN^+^ sEV levels may indicate alternative pathways driving resistance, enabling tailored therapies like sEV biogenesis inhibitors to improve outcomes in this challenging subgroup^[^123^]^.

### Future directions

Multicenter clinical trials are essential to validate PDPN⁺ sEVs and their noncoding RNAs, such as miR-21, as reliable biomarkers for OC, enabling early diagnosis and personalized treatment^[^124^]^. Standardizing EV and noncoding RNA isolation and analytical techniques is critical to ensure reproducibility and facilitate clinical translation^[^125^]^. These standardized protocols will support consistent biomarker detection across diverse patient cohorts.

Therapeutic strategies combining PDPN–CLEC-2 blockade with chemotherapy or poly (ADP-ribose) polymerase inhibitors (PARPi) hold promise for overcoming chemo resistance and reducing tumor dissemination in advanced-stage OC^[^126^]^. Clinical trials evaluating these combination therapies could target tumor–platelet–CSC interactions, enhancing treatment efficacy^[^127^]^. Longitudinal monitoring of PDPN⁺ sEV and miR-21 levels in patient plasma offers a minimally invasive approach to track disease recurrence, particularly in high-risk cases, providing real-time insights into disease progression^[^124^]^.

Artificial intelligence (AI)-driven predictive models integrating liquid biopsy data from PDPN⁺ sEVs and associated noncoding RNAs could forecast therapeutic response or resistance, optimizing treatment strategies^[^128^]^. These models may improve precision medicine by identifying patients likely to benefit from specific therapies. Collaborative efforts to standardize methodologies and validate biomarkers through large-scale studies are imperative to shift OC management from late-stage diagnosis to early, tailored interventions, ultimately improving patient outcomes.

Emerging therapeutic strategies are beginning to explore combinatorial approaches integrating PDPN–CLEC-2 blockade with standard treatments, such as PARP inhibitors, particularly in platinum-resistant OC. Preclinical evidence suggests that disrupting platelets–tumor crosstalk may enhance sensitivity to DNA damage–based therapies, while EV-based delivery systems offer a promising platform for the targeted modulation of non-coding RNAs. Liquid biopsy-guided patient selection may further refine these strategies and accelerate their translation into precision oncology^[^78,115–121^]^.

## Conclusion

OC cells secrete sEVs enriched with PDPN⁺, which significantly contribute to tumor progression through multiple mechanisms. These vesicles activate platelets via interaction with CLEC-2, initiating thrombotic cascades that foster a pro-tumorigenic microenvironment. Additionally, PDPN⁺ sEVs deliver TGF-β and PDGF, promoting CSC proliferation and inducing invasive phenotypic transitions. Furthermore, these vesicles encapsulate noncoding RNAs, including miR-21 and miR-200b, detectable in patient plasma, which hold potential as liquid-biopsy biomarkers for early detection and longitudinal monitoring of OC progression.

Therapeutic strategies targeting PDPN–CLEC-2 interactions or sEV release pathways offer promising avenues to mitigate chemotherapy resistance. Preclinical studies indicate that such interventions effectively reduce metastatic dissemination and thrombosis risk without increasing bleeding complications. These findings elucidate the critical role of PDPN⁺ sEVs in OC biology and underscore their potential as diagnostic and therapeutic targets, paving the way for precision medicine approaches to improve clinical outcomes in OC.

Lastly, this narrative review adheres to the Transparency in the Reporting of Artificial Intelligence in Research (TITAN) guideline^[^129^]^. During the preparation of this manuscript, generative AI tools (DeepSeek, DeepSeek Inc., June 2025 version: temperature parameter = 0.7) were used only as auxiliary aids to assist with formatting, standardization, organization of background information, and language consistency checks. Furthermore, all AI-assisted text was thoroughly reviewed, edited, and refined by the authors. The conceptualization, literature interpretation, synthesis of ideas, and manuscript writing were performed entirely by the authors, ensuring the academic rigor, accuracy, and originality of the review.

## Data Availability

All data discussed in this narrative review are derived from previously published studies, publicly available databases, and cited literature. No new datasets were generated or analyzed for this study.

## Research registration unique identification number (UIN)

Not applicable. This manuscript is a narrative review and does not involve primary clinical research or interventional studies requiring registration.
